# Using Dual Neural Network Architecture to Detect the Risk of Dementia With Community Health Data: Algorithm Development and Validation Study

**DOI:** 10.2196/19870

**Published:** 2020-08-31

**Authors:** Xiao Shen, Guanjin Wang, Rick Yiu-Cho Kwan, Kup-Sze Choi

**Affiliations:** 1 Centre for Smart Health School of Nursing The Hong Kong Polytechnic University Kowloon Hong Kong; 2 Murdoch University Western Australia Australia; 3 School of Nursing The Hong Kong Polytechnic University Kowloon Hong Kong

**Keywords:** cognitive screening, dementia risk, dual neural network, predictive models, primary care

## Abstract

**Background:**

Recent studies have revealed lifestyle behavioral risk factors that can be modified to reduce the risk of dementia. As modification of lifestyle takes time, early identification of people with high dementia risk is important for timely intervention and support. As cognitive impairment is a diagnostic criterion of dementia, cognitive assessment tools are used in primary care to screen for clinically unevaluated cases. Among them, Mini-Mental State Examination (MMSE) is a very common instrument. However, MMSE is a questionnaire that is administered when symptoms of memory decline have occurred. Early administration at the asymptomatic stage and repeated measurements would lead to a practice effect that degrades the effectiveness of MMSE when it is used at later stages.

**Objective:**

The aim of this study was to exploit machine learning techniques to assist health care professionals in detecting high-risk individuals by predicting the results of MMSE using elderly health data collected from community-based primary care services.

**Methods:**

A health data set of 2299 samples was adopted in the study. The input data were divided into two groups of different characteristics (ie, client profile data and health assessment data). The predictive output was the result of two-class classification of the normal and high-risk cases that were defined based on MMSE. A dual neural network (DNN) model was proposed to obtain the latent representations of the two groups of input data separately, which were then concatenated for the two-class classification. Mean and *k*-nearest neighbor were used separately to tackle missing data, whereas a cost-sensitive learning (CSL) algorithm was proposed to deal with class imbalance. The performance of the DNN was evaluated by comparing it with that of conventional machine learning methods.

**Results:**

A total of 16 predictive models were built using the elderly health data set. Among them, the proposed DNN with CSL outperformed in the detection of high-risk cases. The area under the receiver operating characteristic curve, average precision, sensitivity, and specificity reached 0.84, 0.88, 0.73, and 0.80, respectively.

**Conclusions:**

The proposed method has the potential to serve as a tool to screen for elderly people with cognitive impairment and predict high-risk cases of dementia at the asymptomatic stage, providing health care professionals with early signals that can prompt suggestions for a follow-up or a detailed diagnosis.

## Introduction

### Background

Dementia is a collective term referring to a group of diseases that cause a decline in cognitive function owing to brain cell damage. The symptoms include degradation in memory, communication, or reasoning ability, which can seriously interfere with activities of daily living [1]. Dementia is aging related. The incidence doubles with every increase in age of 5.9 years [2], and the number of people living with dementia worldwide is estimated to increase almost three times from 47 million in 2015 to 135 million in 2050 [3]. Thus, dementia is not only an overwhelming issue among elderly people and their families, but also an unprecedented burden on the health social care system and the society at large [4].

It has been reported that 35% of dementia cases are attributable to modifiable risk factors, such as hypertension, obesity, depression, and smoking [5], which concern physical, cognitive, and social inactivity and can be countered through lifestyle interventions [6]. As it takes time to modify lifestyle, early detection of people with high risk of dementia is important to enable timely diagnosis and intervention, which may halt or delay the development of dementia [7-9]. However, underdiagnosis of dementia at the early stage is common since the symptoms are subtle and the progression of cognitive impairment is insidious and cannot be easily observed by the person, family members, or even health care professionals [10,11].

Apart from cognitive symptoms, dementia risk is also associated with many noncognitive conditions (eg, cardiovascular conditions, nutrition, mobility, and depression) [12], which are routinely and vastly obtained from primary care settings, such as elderly community centers. While these routinely collected data provide good potential for the risk prediction of dementia, there is a lack of formulae in the literature to estimate the risk of dementia by using these data.

With the advance of artificial intelligence, machine learning offers a promising approach for the intelligent detection of dementia risk, particularly when the causal connections with risk factors remain unclear. A “school of methods” is to apply machine learning techniques to the data collected from population or community-based settings [13], such as the results of neuropsychology tests or physical examinations, to screen for people with high risk of dementia. While statistical analysis methods like logistic regression and Cox proportional hazard regression are commonly used for analyzing community-acquired elderly health data [14-16], various machine learning techniques have been employed. Among the techniques, supervised machine learning methods represent a majority [17-19], and they include naïve Bayes, decision tree (DT), random forest (RF), artificial neural network, and support vector machine (SVM), whereas their unsupervised counterparts have also been exploited for dementia risk prediction [20]. Nevertheless, missing data is a common problem with data collected from population or community-based settings. Data may be lost owing to noncompliance with appointment schedules, unwillingness to respond to specific questions, or inadvertence of interviewers. Discarding records with missing data and imputation with population means are conventionally used methods to deal with missing data [17,20,21]. Another issue of data analysis is class imbalance, where samples of the target (ie, high-risk cases) and nontarget (ie, normal cases) are disproportionate. When learning from imbalanced data, supervised machine learning algorithms are usually overwhelmed by majority class examples [22]. Other than simply reducing the size of sample-abundant data sets, oversampling methods, such as the synthetic minority oversampling technique, can be used to balance the data sets [23]. In addition, cost-sensitive learning (CSL) is an effective method to handle class imbalance, which is employed in machine learning algorithms to set the cost ratio according to prior class distributions [22,24-27].

In primary care, Mini-Mental State Examination (MMSE) [28] is a commonly used tool for screening cognitive impairment, which is a strong diagnostic criterion of dementia. However, MMSE is a questionnaire that is administered when symptoms of memory decline have occurred, and early administration at the asymptomatic stage or repeated measurements would lead to a practice effect [29] (the questions could be remembered), degrading the effectiveness of MMSE when used at later stages.

### Objective

The aim of this study was to develop an alternative machine learning approach based on MMSE that can be used for screening cognitive impairment and the early detection of people with high dementia risk at the asymptomatic stage. The data adopted were collected through the delivery of elderly care services in the community, which included a wide range of health assessments. A dual neural network (DNN) model was proposed to learn latent representations by utilizing the health profiles of elderly clients and the results of health assessment questionnaires as two types of input features. The predictive output of the model was the result of two-class classification of normal versus high-risk cases, which were defined based on MMSE. Furthermore, the mean and *k*-nearest neighbor (KNN) imputation methods were used to deal with missing data, whereas CSL was used to deal with class imbalance. The performance of the DNN model was evaluated experimentally and compared with that of conventional machine learning algorithms. It was hypothesized that with CSL, the proposed DNN would outperform the algorithms under comparison.

The major contributions of the study are as follows: (1) the community-based health data that were collected for 10 years during elderly care services could provide useful information to meet the increasing emphasis on primary care development (the data set can be shared by request from a qualified researcher; the request should be directed to the corresponding author); (2) the study explored the use of the data set for predicting the risk of dementia, which is a new approach to the best of our knowledge; (3) as the data set has two different characteristics, innovative use of the contemporary DNN architecture was proposed as a new informatics method to fit the specific application scenario; (4) KNN and CSL were incorporated to solve the problems of missing data and class imbalance; and (5) extensive experiments were conducted for comparisons with classical algorithms that are commonly used in health care research to demonstrate outperformance and provide evidence to support the feasibility for dementia risk prediction.

## Methods

### Community Health Data

The data set used was obtained through mobile health care services offered in collaboration with elderly care centers run by local nongovernmental organizations. The health care services were provided for community-dwelling elderly people living in various districts of Hong Kong for free during the period from 2008 to 2018. The services included a wide range of elderly-specific health assessments, where follow-up appointments, workshops, and programs were arranged to promote health care and self-management. The data set included demographic information of elderly clients (eg, gender, age, marital status, type of residency, relationship with roommates, and social participation), bio-measurements (eg, body temperature, pulse rate, oxygen saturation, blood pressure, and waist-hip ratio), and medical history (eg, records of health problems or past diseases), as well as comprehensive information collected using a battery of health assessment questionnaires (ie, MMSE [28], brief pain inventory [BPI] [30], elderly mobility scale [EMS] [31], geriatric depression scale [GDS] [32], mini-nutrition assessment [MNA] [33], constipation questionnaire [CQ] [34], and a questionnaire based on the Roper-Logan-Tierney model of nursing [RLT] [35]), the records of gross oral hygiene and visual acuity assessments, and a survey of the favorite activities of the elderly clients. The health assessment questionnaires will be discussed further in the next section. The elderly health care services were provided by registered nurses and advanced practice nurses or student nurses under supervision, who were also responsible for recording the data while conducting health assessments or administering the questionnaires.

### Health Assessments

The data set adopted contained the results of 10 health assessments, which are described below.

#### Mini-Mental State Examination

MMSE is a quick and reliable assessment of cognitive impairment in older adults. The use of MMSE as part of the process for diagnosing dementia is supported by a Cochrane review of 24,310 citations [36]. MMSE consists of six sets of questions focusing on the cognitive aspects of mental function. For example, elderly clients were asked to give the date of the day, perform arithmetic operations, and perform hand drawing. The assessment can be completed within 10 minutes. The maximum score is 30. A score between 24 and 30 indicates normal cognition, whereas a score below 24 suggests various degrees of impairment, with a lower score indicating greater impairment. In this study, two-class classification was adopted (ie, normal [score ≥24] and high risk [score <24]).

#### Brief Pain Inventory

The BPI is a questionnaire used to assess the severity of pain and its influence on elderly people [30]. The short-form BPI was administered, and it has nine items concerning the location and degree of pain in the last 24 hours, treatments being applied, and their influences on functioning, such as walking ability, mood, and sleep.

#### Elderly Mobility Scale

The EMS is a seven-item assessment tool used to evaluate the mobility of elderly people through functional tests (eg, transition between sitting and lying, gait, timed walk, and functional reach) [31]. The maximum score is 20. A score of 14 or above indicates normal mobility and independent living; a score between 10 and 13 indicates a borderline case; and a score below 10 indicates the necessity of assistance to perform activities of daily living.

#### Geriatric Depression Scale

The GDS is a measure of depression in older adults [32]. The short-form GDS was administered in the clinic. It contains 15 yes or no questions, each carrying one point, on the feelings about and attitudes toward various aspects of life. The maximum score is 15. A score greater than five indicates depression.

#### Mini-Nutrition Assessment

The MNA is a tool used to assess the nutritional status of older people [33]. It is administered in two steps. The short form of MNA (MNA-SF), which has six items with a maximum score of 14, is first used to detect signs of decline in ingestion. The questions concern appetite loss, weight loss, and psychological stress in the last 3 months; mobility; and BMI. A score of 11 or below indicates possible malnutrition, and follow-up with the full MNA is required in the second step. The full MNA consists of 12 items with a maximum score of 16, and it involves further details such as independent living, medication, ulcers, diet, feeding modes, and mid-arm and calf circumferences. The maximum total score of the MNA is 30, with a score below 17 indicating malnourishment.

#### Constipation Questionnaire

The CQ is used to assess the severity of functional constipation [34]. The questionnaire administered contains six items with questions concerning frequency of evacuation, difficulty to evacuate, incomplete evacuation, stool and abdominal symptoms, and medication.

#### RLT-Based Questionnaire

Based on the RLT [35], a questionnaire with 36 items was designed to assess the independence of older adults in 12 categories of activities of daily living, including maintaining a safe environment, communication, breathing, eating and drinking, elimination, washing and cleaning, controlling body temperature, mobilization, working and playing, sleeping, expressing sexuality, and dying. The results of the questionnaire can be used to determine the interventions required to enable elderly people to remain independent in activities of daily living.

#### Gross Oral Hygiene Assessment

The assessment tool consists of 20 items concerning various oral hygiene conditions of elderly clients, including teeth cleansing, tooth decay, tooth mobility, denture use, denture care, missing or remaining teeth, calculus, gum bleeding, and oral candidiasis, with which the corresponding tooth locations and symptoms are recoded.

#### Visual Acuity Assessment

Visual acuity of elderly clients was measured at the mobile clinic. The data collected included the distance at which measurement was performed, the visual aid used, and the results of measurements using the Snellen chart and the chart of the logarithm of the minimum angle of resolution (LogMAR chart).

#### Survey of Favorite Activities

The survey involves binary yes or no questions, each recording a favorite activity of the elderly clients. The questions cover a wide range of over 40 activities (eg, playing chess, watching television, listening to radio, fishing, hiking, calligraphy, dancing, and shopping).

### Data Set

The data set contained the records of 2299 elderly clients, with one record per client. Each record had a total of 567 features that were the inputs of the models. The features originated from demographic data, bio-measurements, and medical history, as well as the data collected from the various health assessment questionnaires described above, except MMSE. The scores of MMSE were utilized to generate the output labels of the models. If the score of an elderly client was lower than 24, the corresponding sample was labeled as a “high-risk case;” otherwise, the sample was labeled as a “normal case.”

As shown in Table 1, among the 567 features, complete values were only available from 96 features for all 2299 records. In addition, 49 features had a data missing rate of no more than 10% (ie, the values for these 49 features were missing in less than 10% of the records). The data missing rate of 140 features was over 60%. Besides, the data set was imbalanced, with 1872 normal cases versus 427 high-risk cases.

**Table 1:** Statistics of the features with missing data.

Percentage of missing data	Number of features
0%	96
1%-9%	49
10%-19%	22
20%-29%	6
30%-39%	97
40%-49%	5
50%-59%	152
60%-69%	140

### KNN Imputation

**Figure 1:**
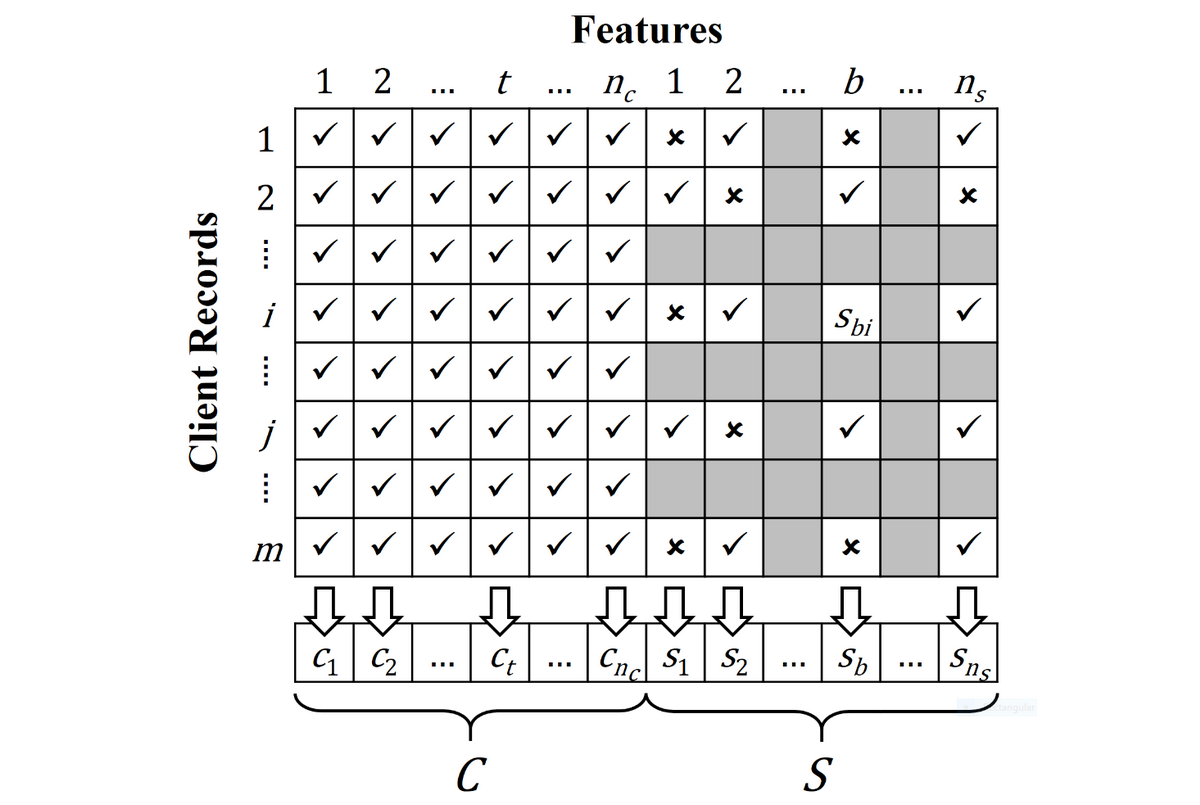
Organization of features into the complete feature set C (left) and the incomplete feature set S (right). The checkmark symbol indicates that a value for a feature is present in a client record, whereas the cross symbol indicates a value is missing (empty).

To address the missing data problem, mean and KNN imputations were used in the study. For mean imputation, the missing values of a client record were filled by the average values of other records with observable feature values. For the KNN imputation method, the missing values of each client record were filled based on the observable values of its KNN. The idea is to assign a higher degree of importance to neighbors that are more similar to the target client record when filling the missing values. With regard to Figure 1, let 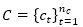 be the set of features with complete values for all records, denoted as *complete features*, and 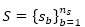 be the set of features with missing values, denoted as *incomplete features*, where *n_c_* and *n_s_* represent the number of complete and incomplete features, respectively. In our data set, *n_c_* was 96 and *n_s_* was 471. Specifically, *c_t_* represents a complete feature where all the client records in the data set have an entry value for the feature *t*. In contrast, *s_b_* indicates an incomplete feature where at least one of the client records in the data set does not provide an entry value for the feature *b*. Furthermore, *s_bi_* represents the entry value of the feature *b* in client record *i*, where *s_bi_* is null if the value of feature *b* in client record *i* is missing. Let *D*∈*R*^m×m^ be a distance matrix that measures the distance between each pair of client records based on all complete features, where *m* is the number of client records in the data set, and *D_ij_* represents the distance between client records *i* and *j*. In this work, we employed Euclidean distance as the distance metric; however, other distance metrics (eg, City Block Distance, Cosine similarity, L1 distance, L2 distance, and Manhattan distance [37,38]) can also be used.

**Figure 2:**
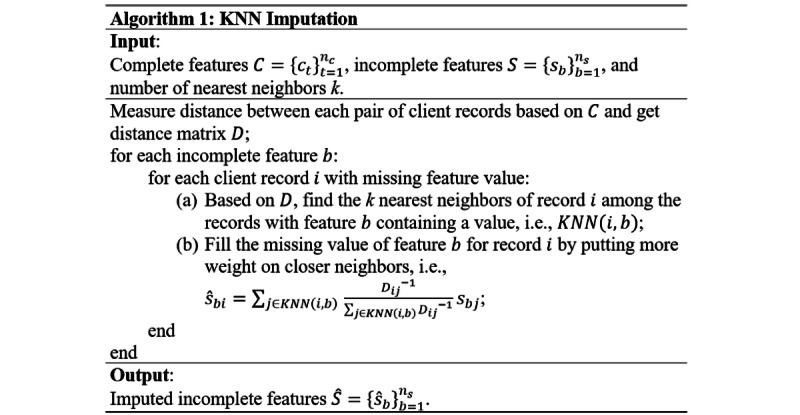
Algorithm of k-nearest neighbor imputation. KNN: k-nearest neighbor.

The algorithm of the KNN imputation method is shown in Figure 2. First, the distance between each pair of client records was measured based on all 96 complete features. Thereafter, the missing values of the incomplete features in a client record were filled with the weighted average of the observable feature values of the *k* nearest records to that client record. After imputation, all the features were treated as “complete” and then utilized as input features of the proposed DNN model for dementia prediction.

### DNN Architecture

In the proposed DNN model, the input features were categorized into two types as follows: client profile and health assessment. The former included demographic information, medical history, and bio-measurements of the elderly clients. The latter included the information collected from nine health assessment questionnaires (ie, BPI, EMS, GDS, MNA, CQ, RLT, gross oral hygiene assessment, visual acuity assessment, and survey of favorite activities).

**Figure 3:**
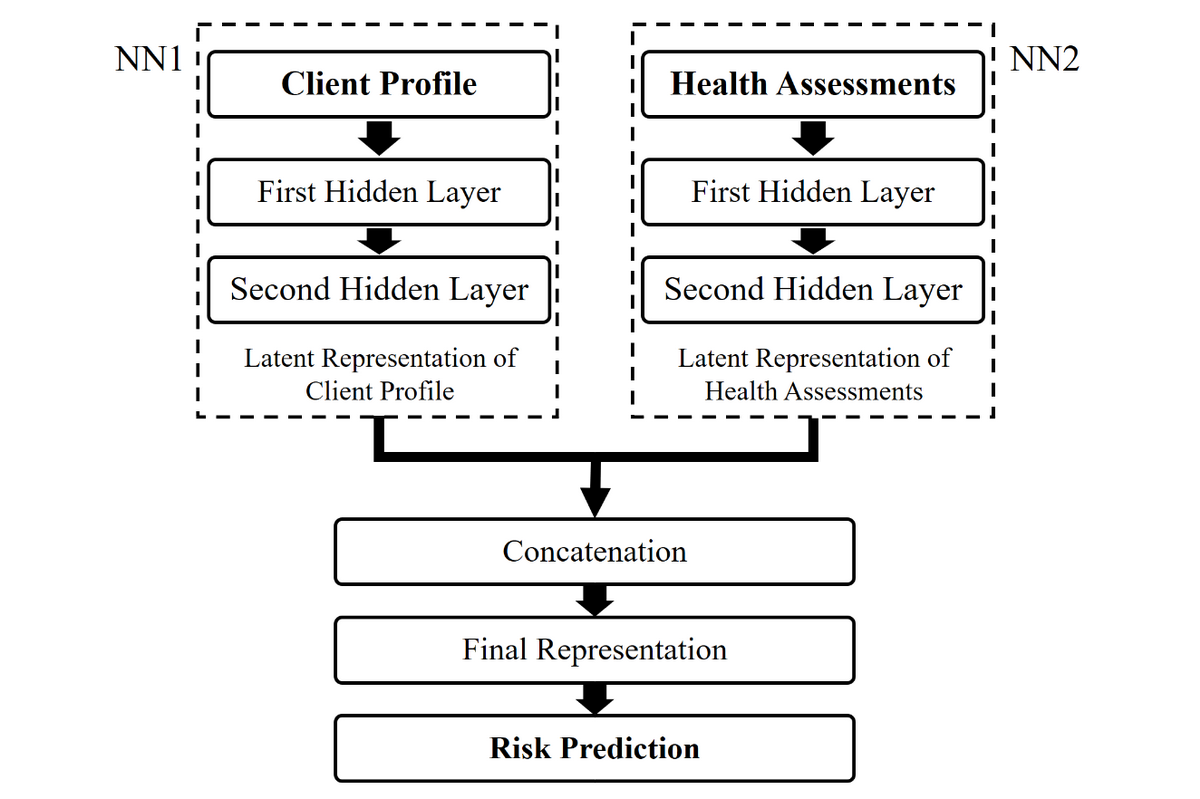
The architecture of the dual neural network. NN1: neural network 1; NN2: neural network 2.

Recently, DNN architecture has been proposed and utilized in state-of-the-art feature representation learning models to learn latent representations based on two types of input features [39-41]. The two types of latent representations are then integrated to learn the final representation for the classification tasks. The approach has demonstrated promising performance in feature representation learning and the ability to capture different kinds of information relevant to the classification task when the two types of input features convey different information and have varied data distributions. Motivated by this approach, we proposed a DNN architecture for screening people with high dementia risk. It learned the latent representations based on the two types of input features concerned in this study. Figure 3 shows the main architecture of the proposed model. With reference to a previous report [40], we employed two neural networks, namely, neural network 1 (NN1) and neural network 2 (NN2), each with two hidden layers, to learn the latent representations for each client from the client profile and health assessments, respectively. The representations were referred to as *latent profile representation* and *latent health assessment representation*. The two latent representations were learned with the two distinct types of features fed as inputs to NN1 and NN2, which were then concatenated to yield the final representation for predicting the dementia risk.

Let *p_i_*∈*R*^1×^*^np^* be a vector representing the profile information associated with client *i*, where *n_p_* is the number of features in the profile. Let *q_i_*∈*R*^1×^*^nq^* be a vector representing the health assessment information associated with client *i*, where *n_q_* is the number of features in the assessment questionnaires. Additionally, *n*=*n_p_*+*n_q_* is the total number of input features. In our data set, *n_p_* was 132, *n_q_* was 435, and *n* was 567.

In NN1, with the client profile information as the input, the latent profile representation was learned layer by layer as follows:


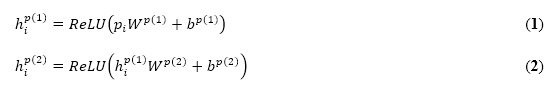


where *ReLU*(⋅)is the rectified linear unit activation function characterized by *ReLU*(*x*)=max(0,*x*), *p_i_* is the input profile feature associated with client *i*, *h_i_^p^*^(1)^∈*R*^1×^*^d^*^1^ and *h_i_^p^*^(2)^∈*R*^1×^*^d^*^2^ represent the latent profile representation of client *i*, learned by the first and second hidden layers of NN1, respectively, and *d*_1_ and *d*_2_ are the dimensionalities of the first and second hidden layers of NN1, respectively. Additionally, *W^p^*^(1)^∈*R^np^*^×^*^d^*^1^ and *b^p^*^(1)^∈*R*^1×^*^d^*^1^ are the trainable weight and bias parameters associated with the first hidden layer of NN1. Moreover, *W^p^*^(2)^∈*R^d^*^1×^*^d^*^2^ and *b^p^*^(2)^∈*R*^1×^*^d^*^2^ are the trainable parameters associated with the second hidden layer of NN1.

Similarly, in NN2, with the information from the health assessment as the input, the latent health assessment representation was learned layer by layer as follows:


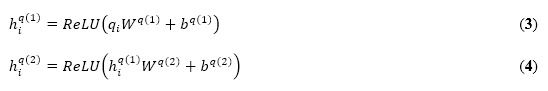


where *q_i_* is the feature of health assessment of client *i* and *h_i_^q^*^(1)^∈*R*^1×^*^d^*^1^ and *h_i_^q^*^(2)^∈*R*^1×^*^d^*^2^ are the latent health assessment representations of client *i* learned by the first and second hidden layers of NN2. Additionally, *W^q^*^(1)^∈*R^nq^*^×^*^d^*^1^, *b^q^*^(1)^∈*R*^1×^*^d^*^1^, *W^q^*^(2)^∈*R^d^*^1×^*^d^*^2^, and *b^q^*^(2)^∈*R*^1×^*^d^*^2^ are the trainable parameters of NN2. In the proposed DNN model, the hidden dimensionalities for NN1 and NN2 were set to be the same.

Thereafter, the deepest latent profile representation learned by NN1 (ie, *h_i_^p^*^(2)^) and the deepest latent health assessment representation learned by NN2 (ie, *h_i_^q^*^(2)^) were concatenated to give the final representation as follows:


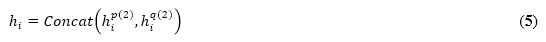


where *h_i_*∈*R*^1×2^*^d^*^2^ is the final representation of client *i*. The final representation is then fed into the classification layer to predict whether an elderly client is high risk or normal as follows:


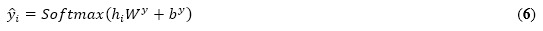


where *ŷ_i_* denotes the predicted probability that client *i* is at high risk. *W^y^* and *b^y^* are the trainable parameters associated with the dementia classification. Given the ground truth labels of the client records that are used as training samples, the supervised classification loss *L* is defined as follows:


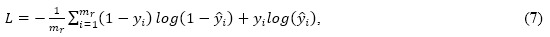


where *m_r_* is the number of training samples. The ground truth label is *y_i_*=1 if the training sample corresponding to client record *i* is a high-risk case and *y_i_*=0 if it is a normal case.

As the data set adopted in the study was imbalanced, with 1872 normal cases and only 427 high-risk cases, the classifiers in supervised machine learning could be biased toward the majority class samples (ie, normal cases). As a screening tool that is used to identify possible cases of high dementia risk, it is important to accurately detect the minority class (high-risk cases). To make the proposed DNN model focus more on high-risk cases, a CSL method was employed by introducing the cost ratio *w* into the classification loss in equation 7 as follows:


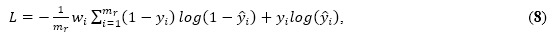


where *w_i_*=*m_r_^n^*⁄*m_r_^d^* if *y_i_*=1 (ie, high-risk case) and *w_i_*=1 if *y_i_*=0 (ie, normal case). Additionally, *m_r_^n^* and *m_r_^d^* are the numbers of normal cases and high-risk cases in the training samples, respectively.

**Figure 4:**
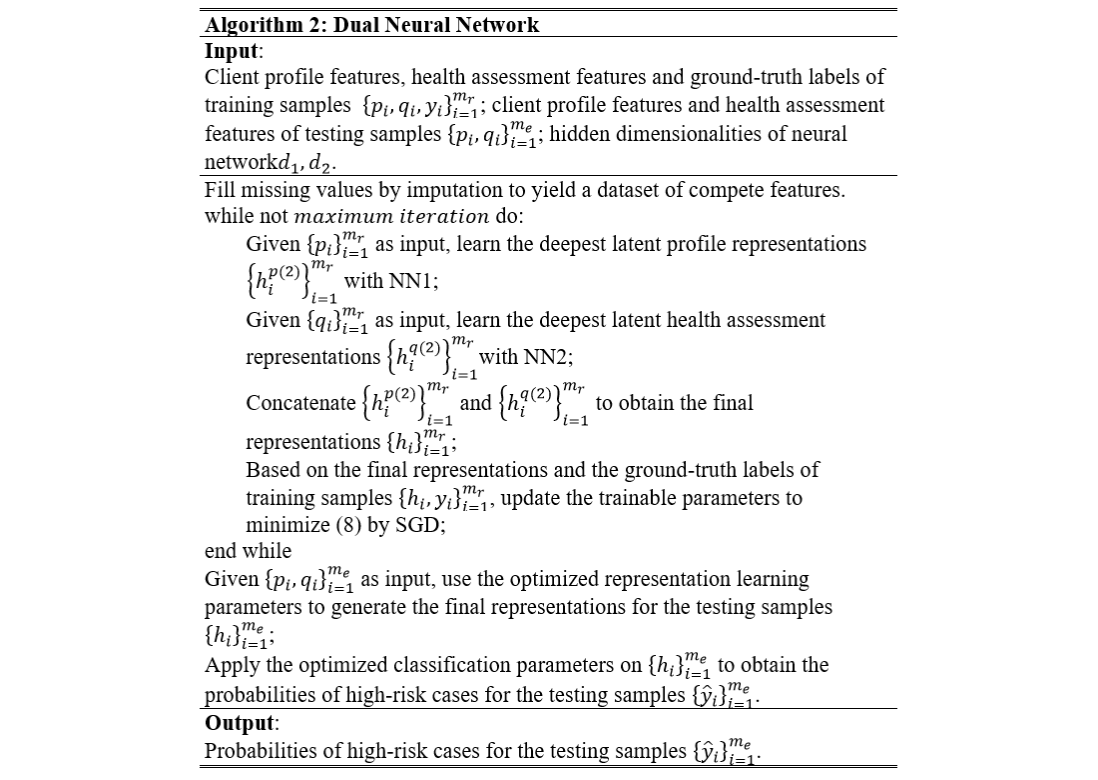
Algorithm of the dual neural network. NN1: neural network 1; NN2: neural network 2; SGD: stochastic gradient descent.

The proposed DNN model was trained following the algorithm shown in Figure 4. First, the missing values in the data set were filled by imputation. For KNN imputation, algorithm 1 was used. Thereafter, NN1 and NN2 were used to learn the latent profile representation and latent health assessment representation, respectively, which were concatenated to yield the final representation for classification. The trainable parameters of NN1 and NN2 that minimize the cost-sensitive classification loss in equation 8 were identified using the stochastic gradient descent (SGD) algorithm [42]. After the model converged, the optimized parameters were employed to generate the final representations and predict the probabilities of high-risk cases for the testing samples.

### Experiments and Settings

The performance of the proposed DNN model was evaluated by making comparisons with five kinds of conventional algorithms (ie, logistic regression [LR], DT, RF, SVM, and single neural network [SNN]). For SVM, three kernel functions were used (ie, linear, polynomial, and radial basis functions, denoted as SVM (linear), SVM (poly), and SVM (RBF), respectively. The SNN, employing all features in one shot as the input, was used to evaluate the effect of introducing an additional neural network in the proposed DNN on classification performance. Moreover, the effect of using CSL to tackle class imbalance was studied by applying it to the algorithms. The corresponding algorithms were denoted as LR+CSL, DT+CSL, RF+CSL, SVM (linear)+CSL, SVM (poly)+CSL, SVM (RBF)+CSL, SNN+CSL, and DNN+CSL. In summary, there were 16 algorithms overall under testing.

In the experiments, mean and KNN imputations were utilized to fill the missing data before model learning. The number of neighbors was set as *k*=5 for the KNN imputation. The LR, DT, RF, and SVM algorithms were implemented using the Scikit-Learn toolkit [43], where default settings were adopted for LR, DT, and the three versions of SVM models with different kernel functions. In RF, the number of trees was set as 100 and the maximum depth of the trees was set as 3. For the DNN, the hidden dimensionalities for both NN1 and NN2 were set with the typical values of *d*_1_=128 and *d*_2_=32. Note that in the DNN, we concatenated the latent representations of NN1 and NN2 as the final representations. To make the SNN and DNN have the same final dimensionality, we set the hidden dimensionalities of SNN as twice of NN1 and NN2 (ie, *d*_1_=256 and *d*_2_=64). All the neural network models were trained by the SGD with a momentum rate of 0.9 following common practice [40]. While normalization to the range of 0 to 1 was initially applied to preprocess the input features, it turned out that the performance degraded instead. Hence, preprocessing methods were not applied in the experiments.

The algorithms under comparison were evaluated with 10-fold cross-validation. The client records were randomly split into 10 folds of equal size. For each of the 10 runs, nine folds of records were employed as training samples and the remaining one fold of records was utilized as testing samples to evaluate prediction performance.

Four performance metrics were adopted, including area under the receiver operating characteristic curve (AUC) [44], average precision (AP) [45], sensitivity, and specificity. For imbalanced data sets, using classification accuracy as an evaluation metric would produce misleading results [46]. Here, AUC was used instead as it is insensitive to class imbalance. The metric AP summarized the precision-recall curve by weighting the precision achieved at each threshold with the increase in recall at the previous threshold. Sensitivity is the recall of high-risk cases (ie, the proportion of “high-risk” testing samples accurately identified). Specificity is the recall of normal cases (ie, the proportion of “normal” testing samples accurately identified).

It was hypothesized that the performance of DNN+CSL would be better than that of the algorithms under comparison, which was tested by running pairwise one-sided *t* tests between DNN+CSL and each algorithm separately in terms of the four metrics. Furthermore, experiments were conducted to investigate variation in the performance of the DNN in terms of AUC and AP with the number of neighbors when KNN imputation was used and with the dimensionalities *d*_1_ and *d*_2_ of the hidden layers in NN1 and NN2.

In addition, the effect of adding fully connected layers (FCLs) between the concatenated representation and the final prediction results was investigated. The experiment was conducted by adding one and two FCLs separately to the proposed DNN+CSL approach and evaluating the performance in terms of the four metrics.

## Results

### Classification Performance

The results of the experiments conducted to evaluate the performance of the algorithms under comparison are shown in Tables 2 and 3, where the mean and SD of the four metrics over 10 runs are provided. In addition, the performance of the proposed DNN+CSL model was compared with that of the other algorithms using a pair-wise *t* test, and the corresponding *P* values are shown in the tables.

As shown in Table 2, when mean imputation was applied, for the metrics AUC and AP, RF+CSL, RF, DNN, and DNN+CSL were the top performing algorithms. For sensitivity, DNN+CSL was among the top three algorithms, with SVM (poly)+CSL and SVM (RBF)+CSL being the first and second algorithms, respectively, and RF exhibited the worst sensitivity (0.01). For specificity, RF, SVM (RBF), and DNN were the top three algorithms. The specificity of DNN+CSL reached 0.80.

Similar results were obtained for KNN imputation. As shown in Table 3, DNN+CSL, DNN, and RF were the top performing algorithms in terms of AUC and AP. DNN+CSL ranked third in sensitivity after SVM (RBF)+CSL and SVM (poly)+CSL. The sensitivity of RF was the worst (0.03). The specificities of RF, SVM (RBF), and DNN were the best and that of DNN+CSL was 0.79.

The results also indicated that the performance of the algorithms evaluated by using mean imputation to tackle missing data was similar to that using KNN imputation. It can also be seen that when CSL was applied to tackle class imbalance, the sensitivity of the algorithms increased and specificity decreased.

**Table 2:** Performance of algorithms with missing data handled by mean imputation.

Algorithm	Mean imputation							
	AUC^a^, mean (SD)	*P* value	AP^b^, mean (SD)	*P* value	Sensitivity, mean (SD)	*P* value	Specificity, mean (SD)	*P* value
LR^c^	0.82 (0.04)	.02	0.87 (0.03)	.047	0.50 (0.10)	<.001	0.91 (0.03)	>.99
LR+CSL^d^	0.82 (0.04)	.02	0.87 (0.03)	.03	0.67 (0.07)	.002	0.82 (0.02)	.98
DT^e^	0.65 (0.05)	<.001	0.76 (0.03)	<.001	0.43 (0.09)	<.001	0.87 (0.03)	>.99
DT+CSL	0.64 (0.02)	<.001	0.75 (0.03)	<.001	0.41 (0.05)	<.001	0.87 (0.02)	>.99
RF^f^	0.84 (0.05)	.52	0.89 (0.03)	.90	0.01 (0.01)	<.001	1.00 (0.00)	>.99
RF+CSL	0.84 (0.05)	.67	0.89 (0.03)	.93	0.64 (0.09)	.001	0.84 (0.03)	>.99
SVM^g^ (RBF^h^)	0.78 (0.06)	<.001	0.85 (0.03)	<.001	0.12 (0.04)	<.001	0.99 (0.01)	>.99
SVM (RBF)+CSL	0.81 (0.05)	<.001	0.86 (0.03)	<.001	0.76 (0.08)	.98	0.73 (0.03)	<.001
SVM (poly^i^)	0.74 (0.06)	<.001	0.83 (0.03)	<.001	0.50 (0.07)	<.001	0.84 (0.03)	>.99
SVM (poly)+CSL	0.81 (0.05)	<.001	0.87 (0.03)	<.001	0.77 (0.08)	.99	0.73 (0.03)	<.001
SVM (linear)	0.79 (0.04)	<.001	0.85 (0.03)	<.001	0.48 (0.07)	<.001	0.89 (0.02)	>.99
SVM (linear)+CSL	0.80 (0.04)	.004	0.86 (0.03)	.005	0.65 (0.07)	<.001	0.81 (0.02)	.94
SNN^j^	0.81 (0.05)	<.001	0.87 (0.03)	.003	0.32 (0.09)	<.001	0.95 (0.01)	>.99
SNN+CSL	0.81 (0.05)	<.001	0.87 (0.03)	<.001	0.65 (0.11)	.002	0.83 (0.02)	>.99
DNN^k^	0.83 (0.05)	.045	0.88 (0.03)	.13	0.33 (0.09)	<.001	0.96 (0.02)	>.99
DNN+CSL	0.84 (0.04)	N/A^l^	0.88 (0.03)	N/A	0.73 (0.09)	N/A	0.80 (0.03)	N/A

^a^AUC: area under the receiver operating characteristic curve.

^b^AP: average precision.

^c^LR: logistic regression.

^d^CSL: cost-sensitive learning.

^e^DT: decision tree.

^f^RF: random forest.

^g^SVM: support vector machine.

^h^RBF: radial basis function kernel.

^i^poly: polynomial kernel.

^j^SNN: single neural network.

^k^DNN: dual neural network.

^l^N/A: not applicable.

**Table 3:** Performance of algorithms with missing data handled by k-nearest neighbor imputation.

Algorithm	KNN^a^ imputation							
	AUC^b^, mean (SD)	*P* value	AP^c^, mean (SD)	*P* value	Sensitivity, mean (SD)	*P* value	Specificity, mean (SD)	*P* value
LR^d^	0.81 (0.04)	.02	0.87 (0.03)	.10	0.46 (0.10)	<.001	0.91 (0.02)	>.99
LR+CSL^e^	0.81 (0.04)	.005	0.87 (0.02)	.03	0.65 (0.09)	.001	0.81 (0.03)	.90
DT^f^	0.67 (0.05)	<.001	0.77 (0.04)	<.001	0.48 (0.09)	<.001	0.86 (0.03)	>.99
DT+CSL	0.66 (0.07)	<.001	0.76 (0.05)	<.001	0.45 (0.13)	<.001	0.86 (0.02)	>.99
RF^g^	0.81 (0.04)	.004	0.87 (0.03)	.13	0.03 (0.04)	<.001	1.00 (0.00)	>.99
RF+CSL	0.81 (0.04)	.004	0.87 (0.03)	.02	0.68 (0.08)	.04	0.78 (0.02)	.10
SVM^h^ (RBF^i^)	0.77 (0.06)	<.001	0.84 (0.03)	<.001	0.08 (0.04)	<.001	0.99 (0.01)	>.99
SVM (RBF)+CSL	0.80 (0.05)	<.001	0.86 (0.03)	<.001	0.75 (0.09)	.90	0.73 (0.02)	<.001
SVM (poly^j^)	0.75 (0.04)	<.001	0.83 (0.03)	<.001	0.50 (0.10)	<.001	0.86 (0.02)	>.99
SVM (poly)+CSL	0.81 (0.05)	<.001	0.86 (0.03)	<.001	0.74 (0.09)	.83	0.73 (0.02)	<.001
SVM (linear)	0.80 (0.04)	.001	0.86 (0.03)	.005	0.48 (0.11)	<.001	0.89 (0.02)	>.99
SVM (linear)+CSL	0.77 (0.04)	<.001	0.85 (0.02)	<.001	0.58 (0.11)	<.001	0.80 (0.02)	.75
SNN^k^	0.81 (0.06)	<.001	0.87 (0.04)	.006	0.33 (0.10)	<.001	0.95 (0.01)	>.99
SNN+CSL	0.80 (0.06)	<.001	0.86 (0.03)	<.001	0.65 (0.11)	.01	0.81 (0.03)	.90
DNN^l^	0.83 (0.05)	.04	0.88 (0.03)	.08	0.35 (0.09)	<.001	0.96 (0.01)	>.99
DNN+CSL	0.84 (0.04)	N/A^m^	0.88 (0.03)	N/A	0.72 (0.10)	N/A	0.79 (0.04)	N/A

^a^KNN: *k*-nearest neighbor.

^b^AUC: area under the receiver operating characteristic curve.

^c^AP: average precision.

^d^LR: logistic regression.

^e^CSL: cost-sensitive learning.

^f^DT: decision tree.

^g^RF: random forest.

^h^SVM: support vector machine.

^i^RBF: radial basis function kernel.

^j^poly: polynomial kernel.

^k^SNN: single neural network.

^l^DNN: dual neural network.

^m^N/A: not applicable.

### Optimal Parameter Setting for the DNN

**Figure 5:**
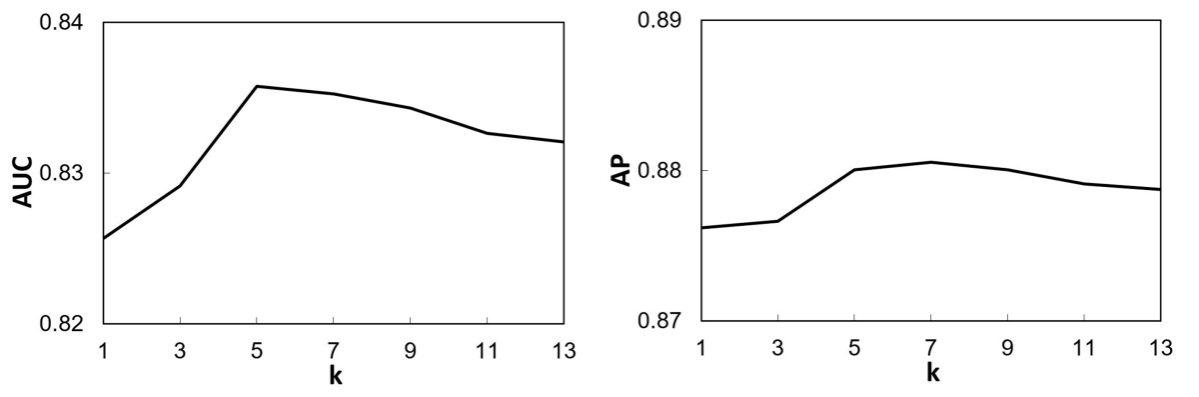
Variation in the area under the receiver operating characteristic curve (AUC) and average precision (AP) with the number of neighbors k in k-nearest neighbor.

**Figure 6:**
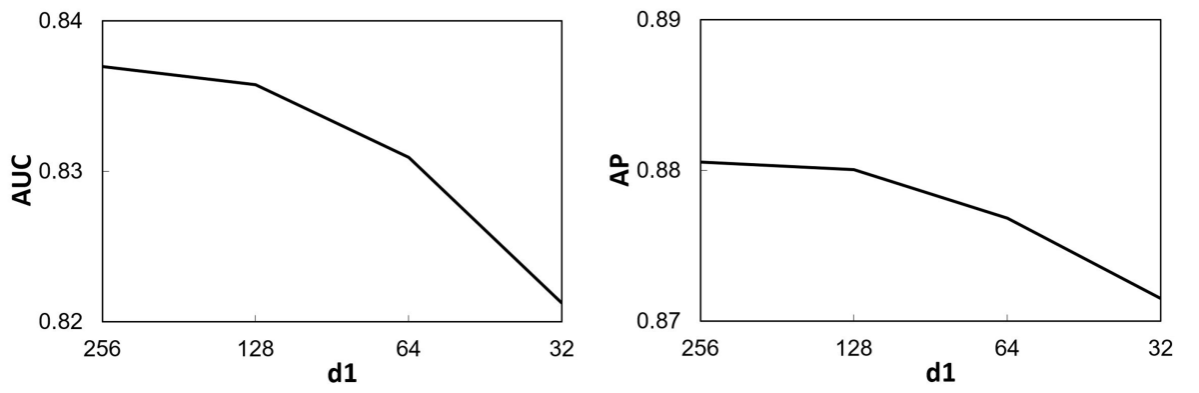
Variation in the area under the receiver operating characteristic curve (AUC) and average precision (AP) with the dimensionality d1 of the first hidden layer in neural network 1 and neural network 2.

**Figure 7:**
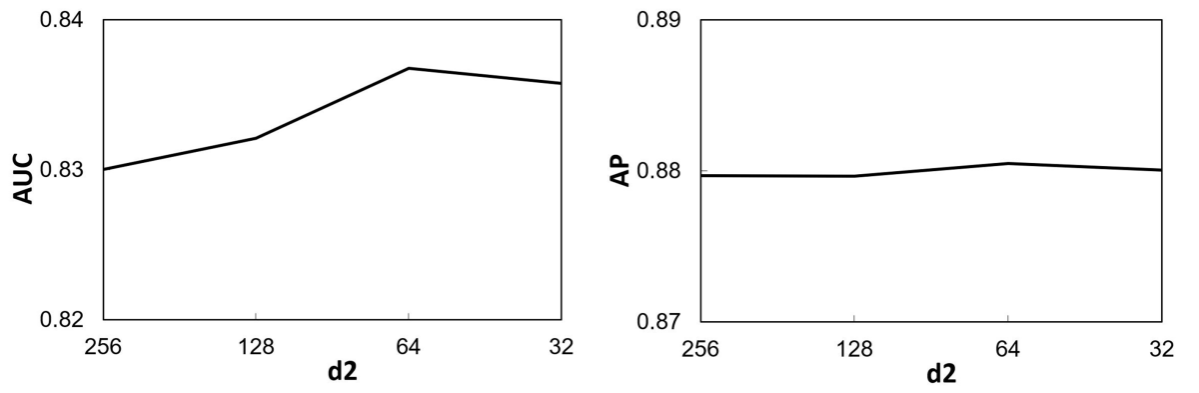
Variation in the area under the receiver operating characteristic curve (AUC) and average precision (AP) with the dimensionality d2 of the second hidden layer in neural network 1 and neural network 2.

The effects of the parameters *k*, *d*_1_, and *d*_2_ on the performance of the proposed DNN in terms of AUC and AP are shown in Figure 5, Figure 6, and Figure 7 respectively. It can be seen from Figure 5 that when KNN imputation was used, both AUC and AP increased with *k* for *k*<5. When *k* was further increased, AUC exhibited a decreasing trend, whereas AP remained at about the same level. This suggests that it is appropriate to set the number of neighbors as *k*=5 for KNN imputation. For the number of dimensions *d*_1_ of the first hidden layer of NN1 and NN2, as shown in Figure 6, a relatively large value (ie, 128 or 256) would yield a higher AUC and AP. In contrast, Figure 7 shows that setting the number of dimensions *d*_2_ of the second hidden layer of NN1 and NN2 to a relatively small value (ie, 64 or 32) would achieve a higher AUC, while AP was insensitive to *d*_2_.

### Effect of FCLs

The effect of adding FCLs to the proposed DNN+CSL model is shown in Table 4. For both mean and KNN imputations, it was found that adding one FCL lowered the AUC and specificity as compared with the finding without an FCL, whereas adding two FCLs lowered the AUC and specificity while increasing sensitivity.

**Table 4:** Effect of fully connected layers on the proposed dual neural network plus cost-sensitive learning model.

Imputation and algorithm		AUC^a^, mean (SD)	AP^b^, mean (SD)	Sensitivity, mean (SD)	Specificity, mean (SD)
**Mean**					
	DNN^c^+CSL^d^	0.84 (0.04)	0.88 (0.03)	0.73 (0.09)	0.80 (0.03)
	DNN+CSL with one FCL^e^	0.83 (0.04)	0.88 (0.03)	0.73 (0.09)	0.79 (0.07)
	DNN+CSL with two FCLs	0.83 (0.05)	0.88 (0.03)	0.77 (0.11)	0.75 (0.04)
**KNN^f^**					
	DNN+CSL	0.84 (0.04)	0.88 (0.03)	0.72 (0.10)	0.79 (0.04)
	DNN+CSL with one FCL	0.83 (0.04)	0.88 (0.03)	0.71 (0.10)	0.77 (0.09)
	DNN+CSL with two FCLs	0.82 (0.05)	0.87 (0.03)	0.77 (0.12)	0.74 (0.03)

^a^AUC: area under the receiver operating characteristic curve.

^b^AP: average precision.

^c^DNN: dual neural network.

^d^CSL: cost-sensitive learning.

^e^FCL: fully connected layer.

^f^KNN: *k*-nearest neighbor.

## Discussion

### Principal Findings

Among the 16 algorithms under testing, DNN+CSL outperformed and consistently ranked among the top three algorithms in terms of AUC, AP, and sensitivity for both mean and KNN imputations. In the case of KNN imputation, DNN+CSL indeed showed the best AUC (mean 0.84, SD 0.04) and AP (mean 0.88, SD 0.03), and ranked third in sensitivity (mean 0.72, SD 0.10). The mean specificity of DNN+CSL reached 0.79 (SD 0.10). Although RF was competitive and ranked among the top three algorithms in terms of AUC, AP, and specificity, the sensitivity was almost zero.

The results suggest that the proposed approach of deep learning with DNNs is promising for screening cognitive impairment in elderly people and thus high-risk cases of dementia. This is attributed to the ability of the DNN to learn hierarchical latent representations from two types of data with different characteristics. The DNN approach is able to capture complex nonlinear relationships between input features and the output.

For both mean and KNN imputations, the performance of using two neural networks in the proposed DNN was much better than that using a SNN in terms of AUC, AP, and sensitivity. While the same features were adopted in both the DNN and SNN, the main difference was that for the DNN, the features were divided into two groups and fed into the two separate neural networks NN1 and NN2. The inputs for NN1 were features concerning the client profile, whereas the inputs for NN2 were features pertaining to the health assessment questionnaires. In the data set adopted, the client profile features were more complete than the health assessment features, that is, more than 72% of the client profile features were complete, while all the features from the health assessment questionnaires contained missing values, with the missing rate ranging from 4.9% to as much as 69.6%. This shows that the elderly clients in general had high acceptance toward the collection of demographic data, information about their medical history, and bio-measurement data, thereby resulting in a low data missing rate for client profile features. On the other hand, the high data missing rate for health assessment features is consistent with the general situation in primary care. According to the frontline health care staff of the clinic, data were missed because clients were absent from scheduled appointments, unable to recall specific events that happened in the past, or declined to respond to questions that they felt uncomfortable to answer or considered sensitive. Given the different characteristics of the two kinds of features, it was beneficial to employ two separate neural networks with different trainable weights to learn the corresponding latent representations.

Furthermore, since all the features were used indiscriminately in the SNN as the input, the characteristics of the two types of features could be interfered or diffused. More importantly, it was likely that the health assessment features, whose quality was affected by missing data, could contaminate the client profile features that were more complete and of better quality. This could be a reason for the suboptimal performance of the SNN as compared with the proposed DNN.

In the data set adopted, the ratio of high-risk cases to normal cases was 1 to 4.4. If the issue of class imbalance was ignored, the classification result would have been biased toward the majority class (ie, normal cases in this study). As a screening tool, high sensitivity is desirable as it is important to identify possible true positives (high-risk cases) and generate early signals, suggesting the potential need for a follow-up. CSL was thus proposed to remedy class imbalance. The effectiveness was evident from the result that the sensitivity of most algorithms improved. For example, when mean imputation was applied, sensitivity increased by 118% for the DNN, 537% for SVM (RBF), and over 70 times for RF, whose sensitivity was almost zero (from 0.01 to 0.64). For missing data imputed using KNN, sensitivity increased by 109% for the DNN, 818% for SVM (RBF), and over 18 times for RF (from 0.03 to 0.68). The increase in sensitivity was achieved at the expense of specificity, with a moderate decrease of less than 26% for data imputed with both imputation methods. Nevertheless, the specificities of the algorithms were still above 0.73 when CSL was applied.

### Limitations

The study presents a machine learning method to screen for elderly people with cognitive impairment and identify high-risk cases of dementia simply by two-class classification. The method can be extended to four-class classification, that is, normal, mild, moderate, and severe, according to MMSE score ranges of 24-30, 19-23, 10-18, and 0-9, respectively. However, the problem of class imbalance would become more relevant. A balanced number of samples for the four classes would be required to construct a fair classification model to avoid predilection for the majority class.

In the proposed DNN architecture, KNN-based imputation was incorporated to tackle missing data, where the nearest neighbors were simply calculated by treating all features with the same weight. Future work will be conducted to design a scheme to assign different weights to different features during KNN imputation.

The elderly health data used in the study were collected from a specific setting of primary care services. Some of the data may not be available from elderly care centers in general, which precludes the use of the proposed DNN-based screening tool. Nevertheless, the client profile data involved and the health assessments adopted were indeed relatively conventional and could be readily integrated into existing health care services in order to make use of the proposed screening tool. On the other hand, future work will be conducted to evaluate and rank the importance of input features, so that less critical features can be dropped to reduce the variety of health data required while still maintaining classification performance.

### Conclusions

This study proposed a DNN approach to screen for elderly people with high risk of dementia using data collected from health care services provided in primary care. Imputation techniques were used to deal with missing data, whereas CSL was adopted to tackle class imbalance. The proposed approach overall outperformed conventional machine learning techniques. It has the potential to serve as an assistive tool for health care professionals to identify people with high risk of dementia at the asymptomatic stage, thereby generating early signals to prompt suggestions for follow-up or the need for a detailed diagnosis of dementia.

## Abbreviations

AP: average precision
AUC: area under the receiver operating characteristic curve
BPI: brief pain inventory
CQ: constipation questionnaire
CSL: cost-sensitive learning
DNN: dual neural network
DT: decision tree
EMS: elderly mobility scale
FCL: fully connected layer
GDS: geriatric depression scale
KNN: k-nearest neighbors
LR: logistic regression
MMSE: Mini-Mental State Examination
MNA: mini-nutrition assessment
NN1: neural network 1
NN2: neural network 2
Poly: polynomial kernel
RBF: radial basis function kernel
RF: random forest
RLT: Roper-Logan-Tierney model of nursing
SGD: stochastic gradient descent
SNN: single neural network
SVM: support vector machine
